# Systemic T-Cell Activation Landscapes Elicited Following Intramuscular and Intranasal Immunization with the Active NDV-Vectored Anti-COVID-19 Vaccine “Patria” in Humans

**DOI:** 10.3390/microorganisms14102232

**Published:** 2026-10-03

**Authors:** Claudia Carranza, Alejandro Torres-Flores, Dámaris P. Romero-Rodríguez, Constantino López-Macías, Martha Torres, Esmeralda Juárez

**Affiliations:** 1 Laboratorio de Inmunobiología de la Tuberculosis, Instituto Nacional de Enfermedades Respiratorias (INER) “Ismael Cosío Villegas”, Mexico City 14080, Mexico; claudia.carranza@iner.gob.mx (C.C.); mtorres@iner.gob.mx (M.T.); 2 Departamento de Biología, Facultad de Química, Universidad Nacional Autónoma de México, Mexico City 04510, Mexico; t.alejandro78@yahoo.com.mx; 3 Laboratorio Nacional Conahcyt de Investigación y Diagnóstico por Inmunocitofluorometría, Instituto Nacional de Enfermedades Respiratorias (INER) “Ismael Cosío Villegas”, Mexico City 14080, Mexico; damaris@iner.gob.mx; 4 Unidad de Investigación Médica en Inmunoquímica, UMAE Hospital de Especialidades, Centro Médico Nacional Siglo XXI, Instituto Mexicano del Seguro Social, Mexico City 06720, Mexico; constantino.lopez135@gmail.com; 5 Laboratorio de Alta Contención Biológica (LACBio), Instituto Nacional de Enfermedades Respiratorias Ismael Cosío Villegas, Mexico City 14080, Mexico

**Keywords:** COVID-19, T-cell responses, Newcastle disease virus-based vaccines, AVX/COVID-12, Intramuscular vaccination, intranasal vaccination

## Abstract

COVID-19 vaccines remain essential for pandemic control, but antibody responses wane, and viral variants emerge. Affordable platforms enabling local manufacturing are important for equitable vaccine access in low- and middle-income countries (LMICs). The locally developed and manufactured NDV-vectored AVX/COVID-12 “Patria” vaccine may help address this need. We conducted an exploratory analysis of antigen-specific cellular immunity in 17 healthy SARS-CoV-2-naïve volunteers from a previously reported phase I safety and immunogenicity cohort (NCT04871737). Eight participants received intramuscular (IM) and nine received intranasal (IN) vaccination. Peripheral blood collected through 90 days was stimulated in vitro with recombinant Wuhan-Spike protein and analyzed using activation-induced marker (AIM) assays, intracellular cytokine staining, and high-dimensional flow cytometry. AIM^+^ CD4^+^ T-cell frequencies peaked around day 28 at 0.45 (1.13)% after IM and 0.18 (0.6)% after IN vaccination, while AIM^+^ CD8^+^ T-cell responses remained lower, reaching 0.09 (0.22)% and 0.03 (0.33)%, respectively, median (IQR). Cytokine-producing T-cell responses showed no consistent route-related differences. High-dimensional analysis identified multiple activation-associated phenotypes in CD3^+^ and CD3^−^ compartments, across both vaccination routes, with distinct temporal dynamics. These exploratory findings show systemic Spike-responsive T-cell activation in this cohort after both routes and provide a refined characterization of vaccine-induced cellular immunity, warranting further investigation of IN administration.

## 1. Introduction

The development and global deployment of anti-COVID-19 vaccines have substantially reduced the incidence of severe disease, hospitalization, and mortality associated with SARS-CoV-2 infection [1]. Although neutralizing antibodies remain an important correlate of protection against infection, increasing evidence indicates that cellular immunity plays a critical role in limiting disease severity, promoting viral clearance, and maintaining long-term immune protection, particularly as viral variants partially evade antibody-mediated neutralization [2,3,4,5]. Antigen-specific CD4^+^ T cells are essential for coordinating adaptive immunity by supporting B-cell maturation, antibody production, and immunological memory, whereas CD8^+^ T cells contribute to the elimination of infected cells and protection against severe disease [2,3,4,5]. Consequently, characterization of vaccine-induced cellular immunity has become increasingly important for understanding the correlates of protection elicited by COVID-19 vaccines.

In addition to vaccine composition, the route of immunization also influences the quality and anatomical distribution of immune responses. Intramuscular (IM) vaccination efficiently induces systemic humoral and cellular immunity, whereas intranasal (IN) vaccination can additionally stimulate immune responses at the respiratory mucosa, the primary site of SARS-CoV-2 entry [6,7,8,9,10]. Experimental and clinical studies have demonstrated that IN immunization can generate tissue-resident immune responses while also eliciting detectable systemic T-cell immunity [6,8,9,10]. Despite growing interest in mucosal vaccination strategies, the phenotypic organization of systemic cellular responses induced by different vaccination routes remains incompletely understood.

The active NDV-vectored anti-COVID-19 vaccine AVX/COVID-12 “Patria” was initially evaluated in a phase I clinical trial involving healthy SARS-CoV-2-naïve volunteers [9]. The vaccine subsequently progressed through phase II (IM and IN booster vaccination) and phase III (IM booster vaccination only) clinical studies, demonstrating a favorable safety profile and non-inferior immunogenicity compared with the ChAdOx1-S vaccine following IM administration, ultimately leading to its approval in Mexico as a booster vaccine for adults [11,12]. The IN booster regimen was also shown to be safe, well-tolerated, and immunogenic, inducing neutralizing antibody titers similar to those elicited by the IM regimen [11]. However, comprehensive analyses of vaccine-induced T-cell responses were performed only in the phase I study, in which two IM doses induced robust serum IgG responses against the SARS-CoV-2 Spike protein together with increased frequencies of IFN-γ-producing T cells, whereas IN vaccination elicited detectable cellular responses despite the absence of measurable systemic antibody responses in SARS-CoV-2-naïve participants [9]. These findings suggested that the route of vaccine administration may influence the qualitative features of vaccine-induced immunity. However, previous immunological analyses were primarily based on cytokine production, leaving the broader phenotypic organization of vaccine-induced cellular activation largely unexplored.

Activation-induced marker (AIM) assays provide a cytokine-independent approach for identifying antigen-responsive T cells following antigen stimulation [13,14]. Combined with recent advances in high-dimensional computational analysis, these approaches enable a more comprehensive characterization of activation-associated cellular phenotypes than conventional manual gating alone [15,16]. Although the primary phase I study established the safety and immunogenicity of AVX/COVID-12 and documented vaccine-induced cellular responses, the magnitude, persistence, and phenotypic organization of systemic antigen-responsive T-cell activation, as well as whether these longitudinal patterns differed between intramuscular (IM) and intranasal (IN) administration, remained to be characterized. Although IN vaccination may offer additional benefits by inducing mucosal immunity, mucosal responses were not directly assessed in the present study, which was restricted to circulating cellular responses. This exploratory secondary analysis therefore focused on systemic cellular responses in the same individuals enrolled in the safety cohort. We aimed to characterize the longitudinal systemic cellular immune responses induced by IM and IN administration of the Patria vaccine through 90 days of follow-up, with particular interest in the persistence and temporal dynamics of responses following intranasal vaccination. Using conventional AIM assays together with unsupervised high-dimensional analysis, we evaluated the dynamics of activation-associated phenotypes within circulating CD3^+^ and CD3^−^ cell compartments following Spike stimulation, providing a more detailed characterization of the systemic cellular responses elicited by both vaccination routes.

## 2. Materials and Methods

### 2.1. Study Groups and Sample Collection

Seventeen healthy SARS-CoV-2-naïve volunteers enrolled in a phase I clinical trial (ClinicalTrials.gov identifier: NCT04871737) evaluating the active NDV-vectored anti-COVID-19 vaccine (AVX/COVID-12) were included in this study [9]. Participants were considered SARS-CoV-2-naïve based on the absence of previous COVID-19 vaccination or known infection, absence of detectable antibodies against SARS-CoV-2 Spike, and a negative SARS-CoV-2 PCR test at enrollment. Participants were randomized to receive two vaccine doses, administered 21 days apart, by either the IM (n = 8) or IN (n = 9) route. Peripheral blood was collected in heparinized tubes (BD Vacutainer, Franklin Lakes, NJ, USA) before vaccination (baseline) and on days 14, 21, 28, 42, and 90 after the first immunization. All participants contributed samples at all reported time points.

### 2.2. Whole Blood Stimulation

Whole blood was diluted 1:1 in RPMI-1640 medium (Lonza, Walkersville, MD, USA) and cultured in the presence of recombinant SARS-CoV-2 Wuhan Spike S1 protein (5 μg/mL; RayBiotech, Peachtree Corners, GA, USA), phytohemagglutinin (PHA, 5 μg/mL; Sigma-Aldrich, St. Louis, MO, USA) as a positive control, or culture medium alone as a negative control. Co-stimulation was performed using anti-human CD28/CD49d antibodies (BD Biosciences, San Jose, CA, USA). Cells were incubated for 18 h at 37 °C under 5% CO_2_. For intracellular cytokine staining, GolgiStop (BD Biosciences) was added during the final 4 h of incubation. Stimulatory concentrations and time were determined in pilot assays, and the same costimulatory conditions were applied to all samples and controls. Following stimulation, cells were stained for viability and processed for flow cytometric analysis.

### 2.3. Flow Cytometry Immunophenotyping and AIM Assay

Following red blood cell lysis, cells were stained with fluorochrome-conjugated antibodies against CD3, CD4, CD8, CD14, CD19, CD56, CD69, CD137, and OX40. Antibody information is summarized in Table S1. For intracellular cytokine staining, cells were fixed and permeabilized using Cytofix/Cytoperm (BD Biosciences, Franklin Lakes, NJ, USA) before staining with antibodies against IFN-γ, TNF-α, and IL-2. For AIM analysis, antigen-responsive CD4^+^ T cells were identified by co-expression of CD69 and OX40, whereas antigen-responsive CD8^+^ T cells were identified by co-expression of CD69 and CD137 following Wuhan Spike protein stimulation. At least 400,000 events were acquired per sample using a BD FACSAria II flow cytometer (BD Biosciences, Franklin Lakes, NJ, USA). Compensation was performed using single-stained controls, and fluorescence-minus-one (FMO) controls were used to define positive populations. Data were analyzed using FlowJo v10.10 (BD Biosciences, Franklin Lakes, NJ, USA). Quality control was initially performed using FlowAI 2.3.2 to identify and remove anomalous events based on signal acquisition stability, fluorescence intensity, and dynamic range [15]. Singlets and lymphocytes were then identified based on light-scatter characteristics, followed by exclusion of dead cells, CD14^+^ monocytes, and CD19^+^ B cells using a LIVE/DEAD/CD14/CD19 dump gate. Live dump-negative lymphocytes were subsequently gated on CD3, followed by CD4/CD8 discrimination and identification of AIM^+^ populations based on CD69/OX40 or CD69/CD137 co-expression. The gating strategy is shown in Figures S1 and S2.

### 2.4. High-Dimensional Analysis of Activation-Associated Phenotypes

Flow cytometry standard (FCS) files corresponding to Spike-stimulated samples were exported from FlowJo for downstream computational analysis in R. CD3^+^ and CD3^−^ compartments were analyzed separately. Quality control was initially performed using FlowAI (Supplementary Figure S1) to identify and remove anomalous events based on signal acquisition stability, fluorescence intensity, and dynamic range [15]. Following quality control, fluorescence intensities for CD4, CD8, CD69, CD137, and OX40 were transformed using an arcsinh transformation with a cofactor of 150. A total of 2000 events were randomly downsampled from each FCS file to prevent samples with higher event counts from disproportionately influencing the unsupervised analysis. Participant identity, vaccination route, sampling time, stimulation condition, and cellular compartment were retained as metadata throughout the analysis. Following downsampling, events were concatenated into a pooled dataset for each cellular compartment. FlowSOM clustering was performed using a 10 × 10 self-organizing map (100 nodes), followed by consensus metaclustering [16]. The final solutions comprised 12 metaclusters for the CD3^+^ compartment and 10 metaclusters for the CD3^−^ compartment. UMAP was generated using the same five transformed markers, with 30 nearest neighbors, a minimum distance of 0.3, Euclidean distance, and a fixed random seed of 123. Metacluster frequencies were calculated separately for each biological sample as the number of events assigned to each metacluster divided by the total number of analyzed events in that sample. Individual flow-cytometry events were used only for dimensionality reduction and clustering and were not considered independent biological replicates; statistical analyses were performed at the participant level. Representative metaclusters were selected post hoc for focused visualization and biological interpretation based on their marker-expression profiles and longitudinal dynamics. Selection was not based on statistical significance or on maximizing differences between vaccination routes. The complete FlowSOM solutions are shown in the global UMAP projections and marker-expression heatmaps.

### 2.5. Statistical Analysis

Continuous variables are presented as the median with the interquartile range unless otherwise indicated. Comparisons between independent groups were performed using the Mann–Whitney U test, whereas paired comparisons were analyzed using the Wilcoxon signed-rank test. Longitudinal changes in AIM responses were evaluated using a mixed-effects model fitted by restricted maximum likelihood (REML), with time, vaccination route, and the time × vaccination route interaction as fixed effects and participant as a random effect. Geisser–Greenhouse correction was applied for repeated measures, and subsequent multiple comparisons were performed using Tukey’s adjustment. Statistical analyses were performed using GraphPad Prism v10 (GraphPad Software, San Diego, CA, USA) and R version 4.5.0 (R Foundation for Statistical Computing, Vienna, Austria). A two-sided *p* value < 0.05 was considered statistically significant.

## 3. Results

### 3.1. Characteristics of the Study Groups

Seventeen healthy, SARS-CoV-2-naïve volunteers enrolled in the AVX/COVID-12 phase I clinical trial (ClinicalTrials.gov identifier: NCT04871737) were included in this study. Only participants assigned to the active vaccine arms were included in the present analysis; individuals originally allocated to the placebo group were not analyzed because they were subsequently offered COVID-19 vaccination in accordance with the trial’s ethical provisions during the pandemic. This cohort size was not powered to detect rare adverse events; rather, it was designed to minimize risk to healthy volunteers while providing sufficient descriptive safety data and dose-escalation boundaries to justify proceeding safely to expanded clinical evaluation. Eight participants received the vaccine by the IM route and nine by the IN route. No statistically significant between-group differences were observed in sex distribution or body mass index, whereas participants in the IM group were younger than those in the IN group (median age, 24.5 vs. 30 years, respectively; *p* = 0.049) (Table 1).

To exclude major differences in circulating immune cell composition before evaluating antigen-induced responses, the frequencies of the main peripheral blood leukocyte subsets were determined at baseline and after completion of the two-dose regimen. No significant differences were observed in circulating CD3^+^, CD4^+^, CD8^+^, CD19^+^, CD3^+^CD56^+^, or CD3^−^CD56^+^ lymphocyte frequencies between the IM and IN groups at baseline or on day 28 (Table 2), indicating that subsequent analyses primarily reflected changes in cellular activation rather than shifts in peripheral blood lymphocyte composition.

### 3.2. AVX/COVID-12 Induces Systemic Antigen-Specific T-Cell Responses Following Intramuscular and Intranasal Administration

**Figure 1:**
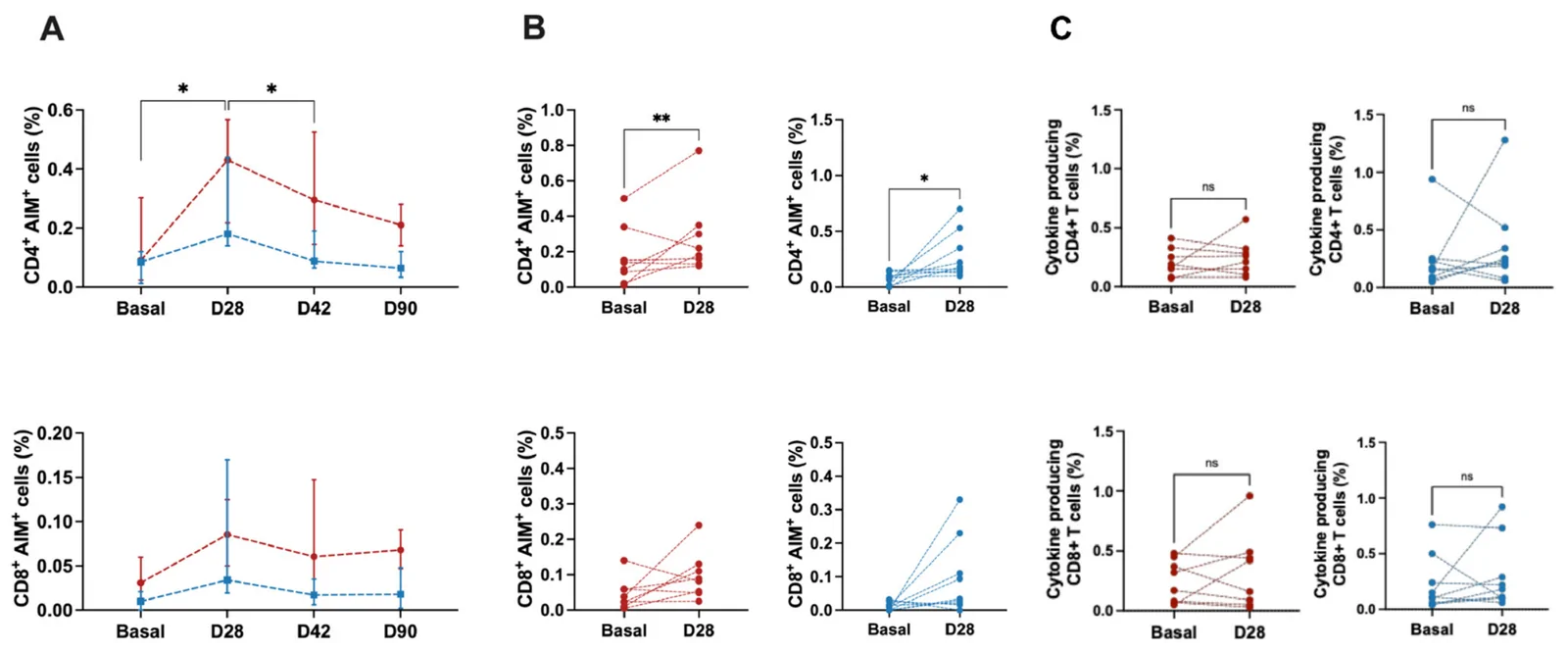
**Intramuscular and intranasal AVX/COVID-12 vaccination induce systemic antigen-specific CD4^+^ and CD8^+^ T-cell responses.** Whole blood samples were stimulated with recombinant SARS-CoV-2 Spike protein for 18 h and analyzed by flow cytometry within live CD3^+^ lymphocytes. AIM^+^ CD4^+^ T cells were defined as CD69^+^OX40^+^ cells, whereas AIM^+^ CD8^+^ T cells were defined as CD69^+^CD137^+^ cells. (**A**) Longitudinal kinetics of Spike-specific AIM^+^ CD4^+^ T cells (**upper panel**) and AIM^+^ CD8^+^ T cells (**lower panel**) in volunteers vaccinated with AVX/COVID-12 through the intramuscular (IM, red) or intranasal (IN, blue) route. Data are presented as median with interquartile range. (**B**) Paired comparison of basal and D28 frequencies of Spike-specific AIM^+^ CD4^+^ T cells (**upper panels**) and AIM^+^ CD8^+^ T cells (**lower panels**) in IM and IN vaccinated individuals. (**C**) Frequencies of cytokines (IFNγ, TNFα, and IL-2)- producing CD4+ T cells (**upper panels**) and CD8+ T cells (**lower panels**). Each line represents an individual participant. Statistical significance was assessed using a mixed-effects analysis followed by Tukey’s multiple comparison test for longitudinal analyses and the Wilcoxon matched-pairs signed-rank test for paired comparisons. * *p* < 0.05; ** *p* < 0.01.

To evaluate systemic antigen-specific T-cell responses induced by AVX/COVID-12 vaccination, peripheral blood cells collected at baseline and throughout follow-up were stimulated in vitro with recombinant SARS-CoV-2 Wuhan Spike protein, and AIM expression was quantified. Spike-responsive CD4^+^ T cells were identified by the co-expression of CD69 and OX40, whereas Spike-responsive CD8^+^ T cells were identified by the co-expression of CD69 and CD137 (Figure 1).

Both vaccination routes induced circulating Spike-responsive CD4^+^ AIM^+^ T cells, with frequencies increasing after vaccination and reaching their highest levels around day 28. In the IM group, CD4^+^ AIM^+^ T-cell frequencies increased significantly compared with baseline at day 28 in the longitudinal mixed-effects analysis, followed by a decline at later time points (Figure 1A). In the IN group, CD4^+^ AIM^+^ T cells also reached their highest frequencies around day 28, although the corresponding increase did not reach statistical significance in the longitudinal mixed-effects analysis. When baseline and day 28 were analyzed separately using the Wilcoxon matched-pairs signed-rank test, a significant increase was detected in both vaccination groups, with median (IQR) frequencies of 0.45% (1.13%) for IM and 0.18% (0.60%) for IN at day 28 (Figure 1B).

Spike-responsive CD8^+^ AIM^+^ T cells were also detected following vaccination, although at lower frequencies than CD4^+^ AIM^+^ T cells. CD8^+^ AIM^+^ T-cell frequencies showed a tendency to increase around day 28 following both vaccination routes (Figure 1A,B).

### 3.3. Systemic Activation-Associated CD3^+^ T-Cell Landscapes Following AVX/COVID-12 Vaccination

**Figure 2:**
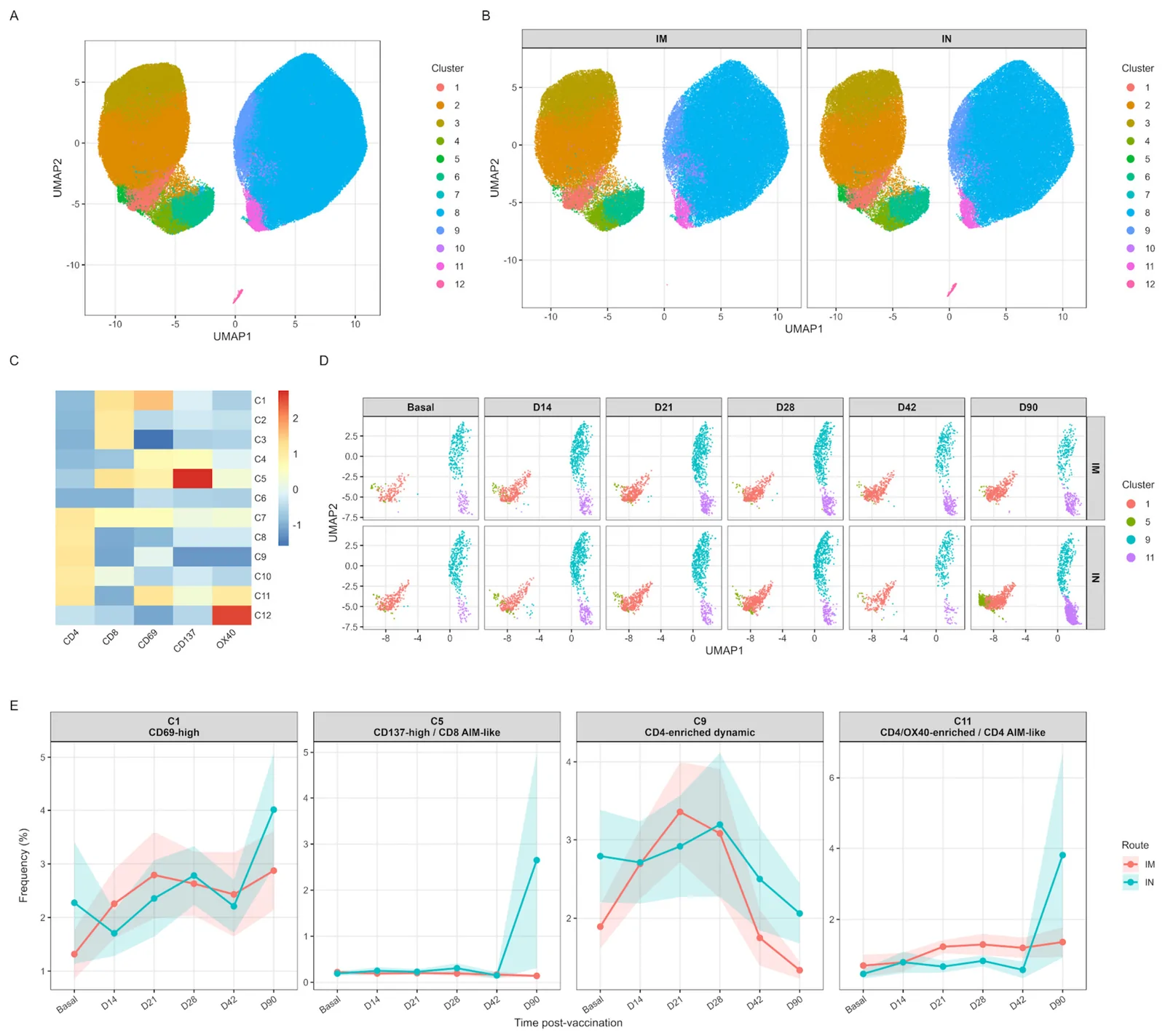
**Intramuscular and intranasal AVX/COVID-12 vaccination elicit similar systemic activation-associated CD3^+^ T-cell landscapes.** (**A**) UMAP projection of all CD3^+^ Spike-stimulated lymphocytes analyzed by FlowSOM, showing the distribution of the 12 identified metaclusters. (**B**) UMAP projection stratified according to vaccination route (intramuscular and intranasal), illustrating the overall distribution of metaclusters in each volunteer’s groups. (**C**) Heatmap showing the median arcsinh-transformed expression (Z-score normalized by marker) of CD4, CD8, CD69, CD137, and OX40 across all FlowSOM metaclusters. Rows represent metaclusters and columns represent phenotypic markers used for clustering. (**D**) UMAP projections displaying the spatial distribution of activation-associated metaclusters (C1, C5, C9, and C11) across vaccination routes and longitudinal sampling time points (Basal, D14, D21, D28, D42, and D90). (**E**) Longitudinal frequencies of representative activation-associated CD3^+^ metaclusters. Data represent mean frequency ± SEM for each vaccination route across study time points. C1 was enriched for CD69 expression, C5 displayed a CD137-high/CD8 AIM-like phenotype, C9 represented a dynamic CD4-enriched population, and C11 exhibited a CD4/OX40-enriched activation-associated phenotype.

To further characterize the phenotypic organization of the systemic Spike-stimulated CD3^+^ T-cell response, high-dimensional unsupervised clustering was performed using CD4, CD8, CD69, CD137, and OX40 expression. Twelve metaclusters were identified and visualized according to vaccination route (Figure 2A,B). Phenotypic characterization of the metaclusters demonstrated similar marker expression profiles across the CD3^+^ compartment. While several metaclusters exhibited minimal expression of activation markers, others showed enrichment for CD69, CD137, and/or OX40, consistent with activation-associated phenotypes that exhibit distinct expression profiles (Figure 2C). Among these, four representative activation-associated metaclusters (C1, C5, C9, and C11) were selected post hoc for longitudinal visualization based on their marker-expression profiles and temporal dynamics (Figure 2D). Descriptively, C1 and C9 showed higher frequencies around days 21–28, whereas C5 and C11 displayed less pronounced temporal variation (Figure 2E).

### 3.4. Systemic Activation-Associated CD3^−^ Cell Landscapes Following AVX/COVID-12 Vaccination

**Figure 3:**
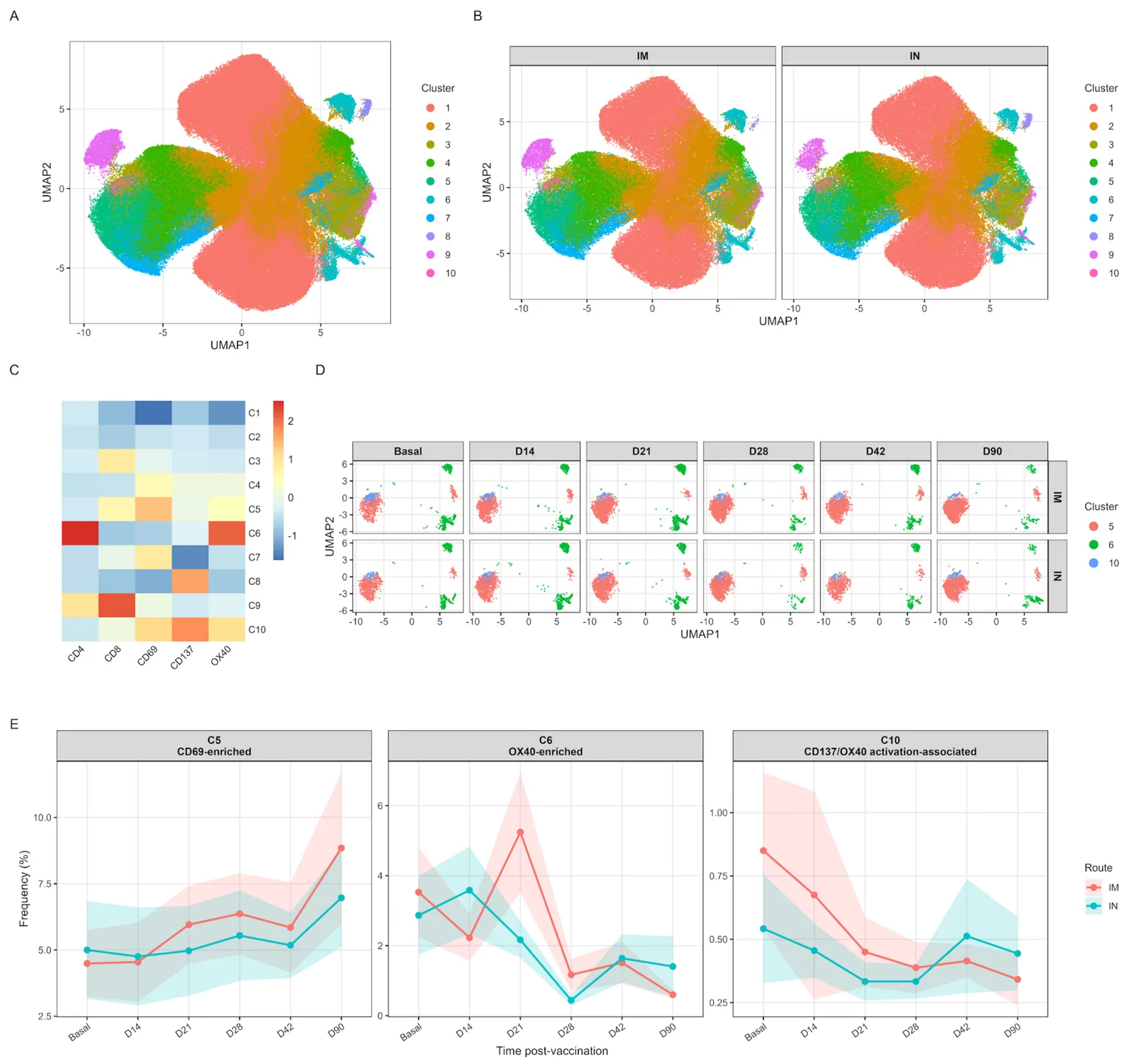
**Similar systemic activation-associated CD3^−^ cell landscapes following intramuscular and intranasal AVX/COVID-12 vaccination.** (**A**) UMAP projection of all CD3^−^ Spike-stimulated cells analyzed by FlowSOM, identifying 10 phenotypically distinct metaclusters. (**B**) UMAP projection separated according to vaccination route (IM and IN), showing the overall distribution of CD3^−^ metaclusters. (**C**) Heatmap of median arcsinh-transformed marker expression (Z-score normalized) for CD4, CD8, CD69, CD137, and OX40 across all CD3^−^ metaclusters. Heatmap colors indicate relative expression levels within each marker. (**D**) UMAP projections highlighting representative activation-associated CD3^−^ metaclusters (C5, C6, and C10) across vaccination routes and longitudinal sampling time points (Basal, D14, D21, D28, D42, and D90). (**E**) Longitudinal frequencies of selected activation-associated CD3^−^ metaclusters. Data are presented as mean frequency ± SEM for each vaccination route throughout follow-up. C5 was enriched for CD69 expression, C6 exhibited an OX40-enriched phenotype, and C10 displayed a CD137/OX40 activation-associated phenotype.

Because activation of circulating CD3^−^ cells was also detected following in vitro Spike stimulation, independently of IFN-γ production (Figure S3), the CD3^−^ compartment was analyzed using the same unsupervised strategy to explore its temporal dynamics after vaccination. Analysis of Spike-stimulated CD3^−^ cells identified 10 metaclusters, which were visualized according to vaccination route (Figure 3A,B). Heatmap visualization demonstrated distinct expression profiles of CD69, CD137, OX40, CD4, and CD8 across CD3^−^ metaclusters, indicating multiple activation-associated phenotypes within this compartment (Figure 3C). Because the available marker set did not allow definitive lineage assignment, these metaclusters were interpreted as CD3^−^ activation-associated phenotypes rather than defined cellular populations. Among these, metaclusters C5, C6, and C10 were selected post hoc for longitudinal visualization based on their activation-associated marker profiles and temporal dynamics (Figure 3D). Descriptively, the OX40-enriched C6 metacluster showed higher frequencies around day 21, particularly following IM vaccination, whereas C5 and C10 displayed less pronounced temporal variation throughout follow-up (Figure 3E).

## 4. Discussion

Although neutralizing antibodies remain an important correlate of protection against SARS-CoV-2 infection, accumulating evidence indicates that cellular immunity plays a fundamental role in limiting disease severity, promoting viral clearance, and maintaining long-term protection, particularly as viral variants partially evade antibody-mediated neutralization [1,17,18,19]. Antigen-specific T-cell responses have therefore emerged as an essential component of vaccine-induced immunity, complementing humoral responses by sustaining immune protection even when circulating antibody titers decline or viral escape variants reduce neutralizing activity [18,19]. In this context, the present study extends previous immunogenicity analyses of the AVX/COVID-12 vaccine by providing a comprehensive longitudinal characterization of systemic cellular activation following both IM and IN immunization.

Activation markers such as CD69, OX40, and CD137 are transiently expressed following antigen recognition and therefore reflect dynamic stages of lymphocyte activation rather than stable differentiation states. Consequently, AIM assays provide a broader assessment of antigen-specific T-cell responses by identifying activated cells independently of their immediate cytokine-producing capacity [13,14]. This feature is particularly relevant because vaccine-induced T-cell responses comprise a continuum of activation, proliferation, differentiation, and memory development that is not necessarily captured by intracellular cytokine staining alone. By combining conventional AIM assays with high-dimensional immunophenotyping, the present study characterized these activation-associated states with greater phenotypic resolution while avoiding reliance on a single functional readout.

The predominance of CD4^+^ AIM responses observed in this study is biologically consistent with the central role of helper T cells during vaccine-induced adaptive immunity. In addition to supporting germinal center formation, affinity maturation, and antibody production, activated CD4^+^ T cells coordinate cytotoxic responses and contribute to the establishment and maintenance of long-lived immunological memory [4,5,20]. Likewise, CD8^+^ T cells contribute to the elimination of infected cells and are considered an important component of protection against severe COVID-19 [4,5,20]. Although the present study was not designed to directly evaluate memory differentiation, the transient increase in activation-associated responses followed by their progressive contraction is consistent with the expected kinetics of antigen-specific T-cell responses following prime-boost vaccination [20,21]. During this process, activated lymphocytes undergo clonal expansion and differentiation into effector populations before contraction and the establishment of long-lived memory subsets [21]. However, because our immunophenotyping panel focused on activation rather than differentiation markers, the development or phenotype of memory T-cell populations could not be directly assessed in this study. Future studies incorporating markers such as CCR7, CD45RA, CD45RO, CD27, CD127, and TCF-1 will be important to address this question.

The biological relevance of these responses may extend beyond the primary vaccination series. Primary vaccination establishes antigen-specific cellular immunity that may contribute to the development of hybrid immunity following subsequent exposure to SARS-CoV-2. Repeated antigen exposure through vaccination, natural infection, or both is associated with broader and more durable T-cell responses capable of recognizing antigenically divergent SARS-CoV-2 variants [19,21]. Although all volunteers included in this phase I trial were SARS-CoV-2-naïve at enrollment, the activation-associated T-cell responses observed following vaccination may represent part of the cellular immune foundation upon which broader responses develop during subsequent antigenic encounters. In this context, understanding how these activation-associated phenotypes evolve following SARS-CoV-2 infection may provide valuable insight into the development and durability of hybrid cellular immunity.

Systemic cellular activation was detected following both IM and IN vaccination. These findings should not be interpreted as evidence that both routes induce equivalent mucosal immunity, since the present study evaluated only circulating immune cells. Importantly, the systemic responses described here do not exclude the possibility that the two routes generate distinct immune landscapes within the respiratory mucosa. Experimental and clinical studies have demonstrated that IN vaccination can preferentially promote tissue-resident immune populations and local antiviral immunity [6,8,22]. Future studies incorporating nasal lavage, bronchoalveolar lavage, or tissue-derived lymphocytes will be required to determine how the systemic activation-associated phenotypes identified here relate to local mucosal immune responses.

Although only one participant in the IN group was older than 40 years and the remaining participants were younger than 37 years, age-related changes in immune function, particularly within T-cell compartments, can begin to emerge during adulthood [23]. Therefore, the baseline age imbalance between groups cannot be excluded as a potential contributor to the observed immune-response patterns.

An additional contribution of this study is the use of high-dimensional computational analysis to complement conventional AIM assays. Rather than defining previously unrecognized immune cell populations, the activation-associated phenotypes identified by unsupervised clustering most likely represent different stages or intensities of cellular activation that coexist during the evolving adaptive immune response. Activation markers such as CD69, CD137, and OX40 reflect transient functional states rather than stable lineage commitment, suggesting that the metaclusters identified in this study represent snapshots of an evolving immunological process rather than discrete biological entities [24]. Consequently, high-dimensional analysis should be viewed as a complementary strategy that increases phenotypic resolution while avoiding assumptions regarding definitive cellular identity.

The biological interpretation of the CD3^−^ compartment deserves particular consideration. Because the antibody panel was designed to characterize activation rather than lineage identity, the cellular composition of these CD3^−^ activation-associated phenotypes cannot be established from the present study. Transient downregulation of surface CD3 following T-cell activation represents one possible explanation [25,26], whereas non-T-cell populations may also contribute to this compartment. In this context, SARS-CoV-2 components can engage innate immune-sensing pathways through different mechanisms. Viral ssRNA motifs have been shown to activate TLR7/8-dependent signaling [27], whereas recombinant SARS-CoV-2 Spike protein, including the S1 subunit, has been reported to induce inflammatory responses in macrophages through TLR2/MyD88-dependent NF-κB signaling [28]. Therefore, although the present study was not designed to define the mechanism responsible for CD3^−^ cell activation, these previously described mechanisms provide plausible biological contexts in which SARS-CoV-2 components, including Spike, may engage innate immune-sensing pathways in parallel with adaptive T-cell activation.

Overall, this study demonstrates that the active NDV-vectored anti-COVID-19 vaccine AVX/COVID-12 “Patria” elicited detectable systemic cellular immune responses following both IM and IN immunization. More importantly, it illustrates how combining conventional AIM assays with high-dimensional immunophenotyping can provide a more comprehensive characterization of vaccine-induced cellular activation. Future studies integrating expanded immunophenotyping, functional assays, and mucosal sampling will be essential to determine the biological relevance and longer-term evolution of these activation-associated phenotypes.

## 5. Limitations

Several limitations should be acknowledged. The relatively small sample size inherent to a phase I clinical trial limits statistical power to detect subtle differences between vaccination routes and precludes robust adjustment for potential confounders. In particular, participants in the IM group were younger than those in the IN group, and this baseline age imbalance may have influenced the observed immune responses. No formal equivalence or non-inferiority analysis between vaccination routes was performed; therefore, the absence of statistically significant route-associated differences should not be interpreted as evidence of equivalence. Immune responses were evaluated exclusively in peripheral blood, precluding direct assessment of tissue-resident or mucosal immune populations. In addition, stimulation was performed using the ancestral Wuhan Spike S1 protein, and therefore the present analysis does not directly assess the breadth of T-cell recognition against currently circulating SARS-CoV-2 variants. The high-dimensional metacluster analysis was exploratory and primarily descriptive, and the longitudinal patterns of selected metaclusters should therefore be considered hypothesis-generating. Moreover, because the marker panel was optimized to characterize activation rather than lineage identity, the cellular identity of the CD3^−^ activation-associated phenotypes cannot be definitively established. Finally, the present study was designed to characterize cellular immunogenicity and did not directly assess clinical protection; therefore, the cellular responses described here should not be interpreted as direct evidence of protective efficacy.

## 6. Conclusions

AVX/COVID-12 “Patria” administered by either the IM or IN route elicited detectable circulating Spike-responsive T-cell activation in this small phase I cohort. High-dimensional computational analysis complemented these findings by providing a refined characterization of activation-associated phenotypes within both the CD3^+^ and CD3^−^ compartments, without implying the existence of route-specific or previously unrecognized immune cell populations. Future studies integrating mucosal sampling, expanded lineage-specific immunophenotyping, and functional assays will be essential to determine how these systemic activation-associated phenotypes relate to local immune responses and their longer-term evolution following NDV-vectored vaccination.

## Figures and Tables

**Table 1:** Demographic and clinical characteristics of the study groups.

Characteristic	IM Scheme n = 8	IN Scheme n = 9	*p*-Value
Age, years, median (range)	24.5 (19–31)	30 (23–46)	^a^ 0.049
Sex, female, n (%)	4 (50)	4 (44.4)	^b^ ns
BMI, index, median (range)	23.3 (20.5–26.3)	23.3 (20.4–26.2)	^a^ ns

Note: IM = Intramuscular administration; IN = Intranasal administration; ns = Not significant. ^a^ Statistical analysis was performed using the Mann–Whitney U test. ^b^ Fisher’s exact test.

**Table 2:** Baseline and day 28 cellular immunophenotyping in volunteers vaccinated with AVX/COVID-12.

Characteristic, Median % (Range)	IM Scheme n = 8	IN Scheme n = 9	*p*-Value
Baseline			
CD3^+^	51.71 (44.96–67.40)	58.17 (5.79–61.58)	ns
CD3^+^CD4^+^	55.99 (48.78–68.39)	60.82 (50.64–73.80)	ns
CD3^+^CD8^+^	35.18 (26.83–44.68)	34.14 (22.19–41.16)	ns
CD3^+^CD56^+^	2.85 (1.15–4.99)	3.72 (0.24–6.65)	ns
CD3^−^CD56^+^	6.80 (5.17–21.37)	6.10 (2.05–30.95)	ns
CD19^+^	13.57 (5.12–20.25)	10.83 (1.59–18.89)	ns
Day 28			
CD3^+^	49.83 (22.09–72.72)	46.57 (17.73–70.42)	ns
CD3^+^CD4^+^	55.49 (48.21–74.59)	57.09 (45.07–70.29)	ns
CD3^+^CD8^+^	37.99 (22.50–43.09)	36.46 (21.80–46.26)	ns
CD3^+^CD56^+^	2.50 (0.99–6.82)	5.14 (0.75–8.26)	ns
CD3^−^CD56^+^	6.10 (2.97–11.65)	7.90 (3.59–22.06)	ns
CD19^+^	10.03 (5.83–25.31)	12.13 (10.29–17.83)	ns

Note: IM = Intramuscular administration; IN = Intranasal administration; ns = Not significant. Statistical analysis was performed using the Mann–Whitney U test.

## Data Availability

The original contributions presented in this study are included in the article/Supplementary Material. Further inquiries can be directed to the corresponding authors.

## Supplementary Materials

The following supporting information can be downloaded at https://www.mdpi.com/article/10.3390/microorganisms14102232/s1; Table S1: List of antibodies used for flow cytometry staining. Figure S1: Flow cytometry quality control and gating strategy. Raw flow cytometry data were initially processed using the FlowAI algorithm to identify and remove anomalous events based on acquisition stability, flow rate, and fluorescence signal consistency, thereby retaining high-quality events for downstream analysis (a). Subsequently, sequential gating was applied to select singlets and lymphocytes based on light-scatter properties, and live cells by excluding Live/Dead^+^ events. The remaining lymphocytes were then separated into CD3^+^ and CD3^−^ compartments for subsequent immunophenotypic and computational analyses (b); Figure S2: Gating strategy and preprocessing workflow for high-dimensional analysis. Flow cytometry data were initially subjected to quality control using the FlowAI algorithm to remove anomalous events. High-quality events from all samples were subsequently processed through sequential gating to select time-stable events, singlets, and lymphocytes based on light scatter properties. Dead cells, along with CD14^+^ and CD19^+^ cells, were excluded using a dump gate, and the remaining lymphocytes were separated into CD3^+^ and CD3^−^ compartments for downstream computational analysis. The resulting populations were exported for UMAP visualization and FlowSOM clustering; Figure S3: Representative gating strategy and conventional AIM analysis of Spike-responsive CD3^+^ and CD3^−^ lymphocytes following AVX/COVID-12 vaccination. Whole blood cells were stimulated in vitro with recombinant SARS-CoV-2 Wuhan Spike protein or culture medium alone for 18 h, and activation-induced marker (AIM) expression was evaluated by flow cytometry. (A,B) Representative gating strategy used to identify AIM^+^ CD4^+^ T cells (CD69^+^OX40^+^), AIM^+^ CD8^+^ T cells (CD69^+^CD137^+^), and activation-associated CD3^−^ cells following intramuscular (IM) (A) or intranasal (IN) (B) vaccination. Frequencies of Spike-responsive AIM^+^ CD4^+^, AIM^+^ CD8^+^, and activation-associated CD3^−^ cells (C,D) and IFNγ-producing cells (F,G) were measured 28 days after IM (C,F, n = 8) or IN (D, G, n = 9) vaccination. Data are presented as box plots showing the median and interquartile range, or as before-after comparisons at 28 days post-vaccination. Statistical comparisons were performed using the Mann–Whitney U test. * *p* < 0.05; ** *p* < 0.01.
