# Supplementary material for: Systemic T-Cell Activation Landscapes Elicited Following Intramuscular and Intranasal Immunization with the Active NDV-Vectored Anti-COVID-19 Vaccine “Patria” in Humans
Source: Microorganisms. 2026 Oct 3;14(10):2232. doi: 10.3390/microorganisms14102232 (PMC13707778; doi:10.3390/microorganisms14102232)
Supplement: Supplementary file 1 [file microorganisms-14-02232-s001.zip › microorganisms-4534511-supplementary.pdf]

# Systemic T-Cell Activation Landscapes Elicited Following Intramuscular and Intranasal Immunization with the Active NDV-Vectored Anti-COVID-19 Vaccine "Patria" in Humans

**Claudia Carranza [1,†], Alejandro Torres-Flores [2,†], Dámaris P. Romero-Rodríguez [3], Constantino López-Macías [4], Martha Torres [1] and and Esmeralda Juárez [5,*]**

## Supplementary material

**Table S1.** List of antibodies used for flow cytometry staining.

| *Antibody against* | *Fluorochrome* | *Supplier* | *Cat. number* | *Dilution (ml)* | *RRID* |
|---|---|---|---|---|---|
| CD3 | AF700 | Biolegend | 344821 | 1:33 | AB_2563419 |
| CD4 | PerCPCy5.5 | Biolegend | 300530 | 1:100 | AB_893322 |
| CD8 | BV605 | Biolegend | 300936 | 1:33 | AB_2832575 |
| CD8 | PE/Cy7 | Biolegend | 980910 | 1:100 | AB_2876774 |
| CD16 | BV510 | Biolegend | 302048 | 1:100 | AB_2562085 |
| CD19 | APC-Cy7 | Biolegend | 302218 | 1:100 | AB_314248 |
| CD19 | APC | BD | 555415 | 1:40 | AB_398597 |
| CD14 | APC-Cy7 | Biolegend | 325620 | 1:100 | AB_830693 |
| CD14 | PE CF594 | BD | 562335 | 1:100 | AB_11153663 |
| CD56 | PE | Biolegend | 362508 | 1:100 | AB_2563925 |
| CD69 | BV421 | Biolegend | 310930 | 1:25 | AB_2561909 |
| CD137 | APC | Biolegend | 309810 | 1:33 | AB_830672 |
| OX40 | PE-Cy7 | Biolegend | 350012 | 1:100 | AB_10901161 |
| IFN-γ | FITC | BD | 554551 | 1:200 | AB_395473 |
| TNF-α | APC | Biolegend | 502912 | 1:33 | AB_315264 |
| IL-2 | BV605 | Biolegend | 500332 | 1:40 | AB_2563877 |

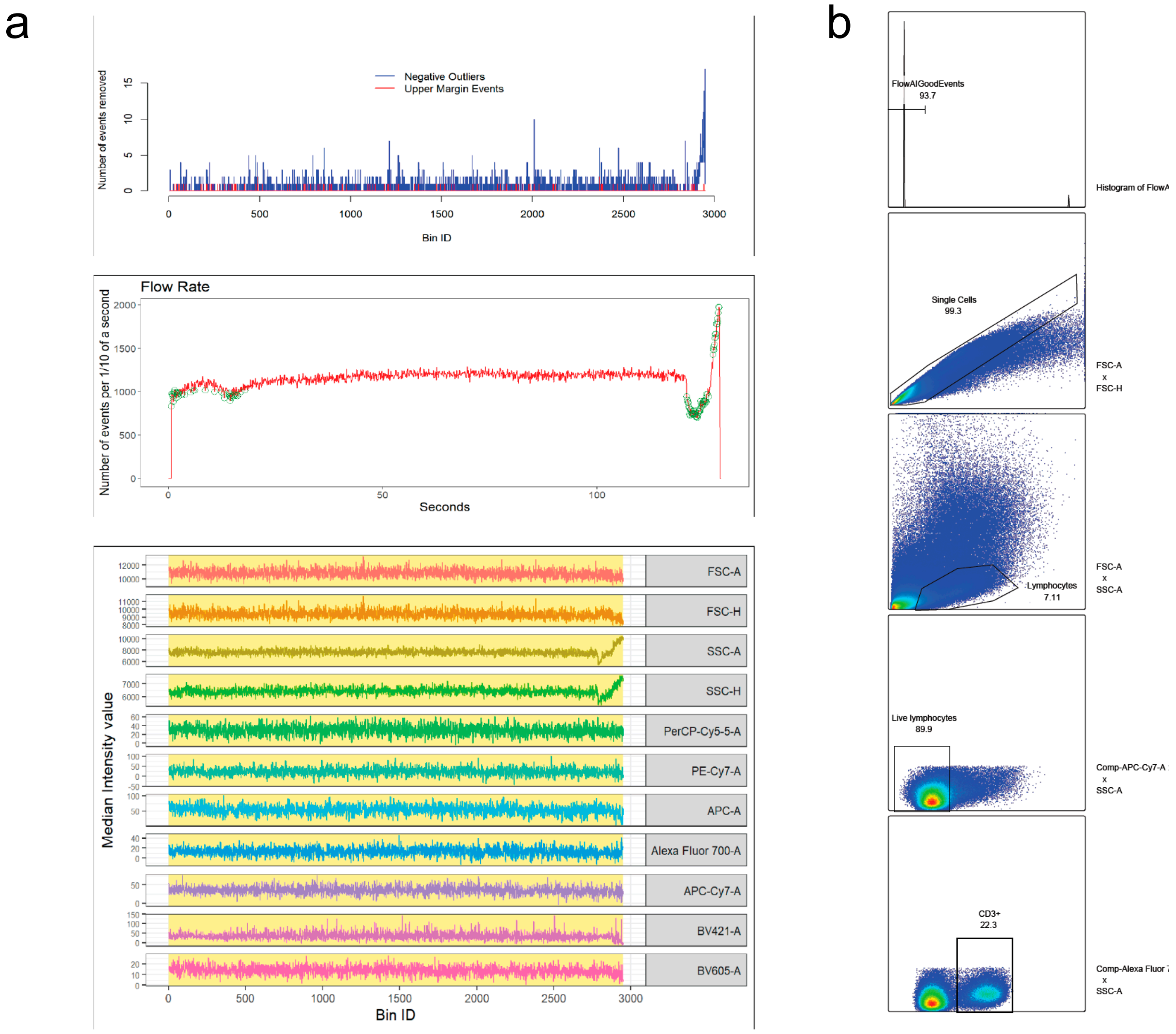


**Figure S1. Flow cytometry quality control and gating strategy.** Raw flow cytometry data were initially processed using the FlowAI algorithm to identify and remove anomalous events based on acquisition stability, flow rate, and fluorescence signal consistency, thereby retaining high-quality events for downstream analysis (a). Subsequently, sequential gating was applied to select singlets and lymphocytes based on light-scatter properties, and live cells by excluding Live/Dead⁺ events. The remaining lymphocytes were then separated into CD3⁺ and CD3⁻ compartments for subsequent immunophenotypic and computational analyses (b).

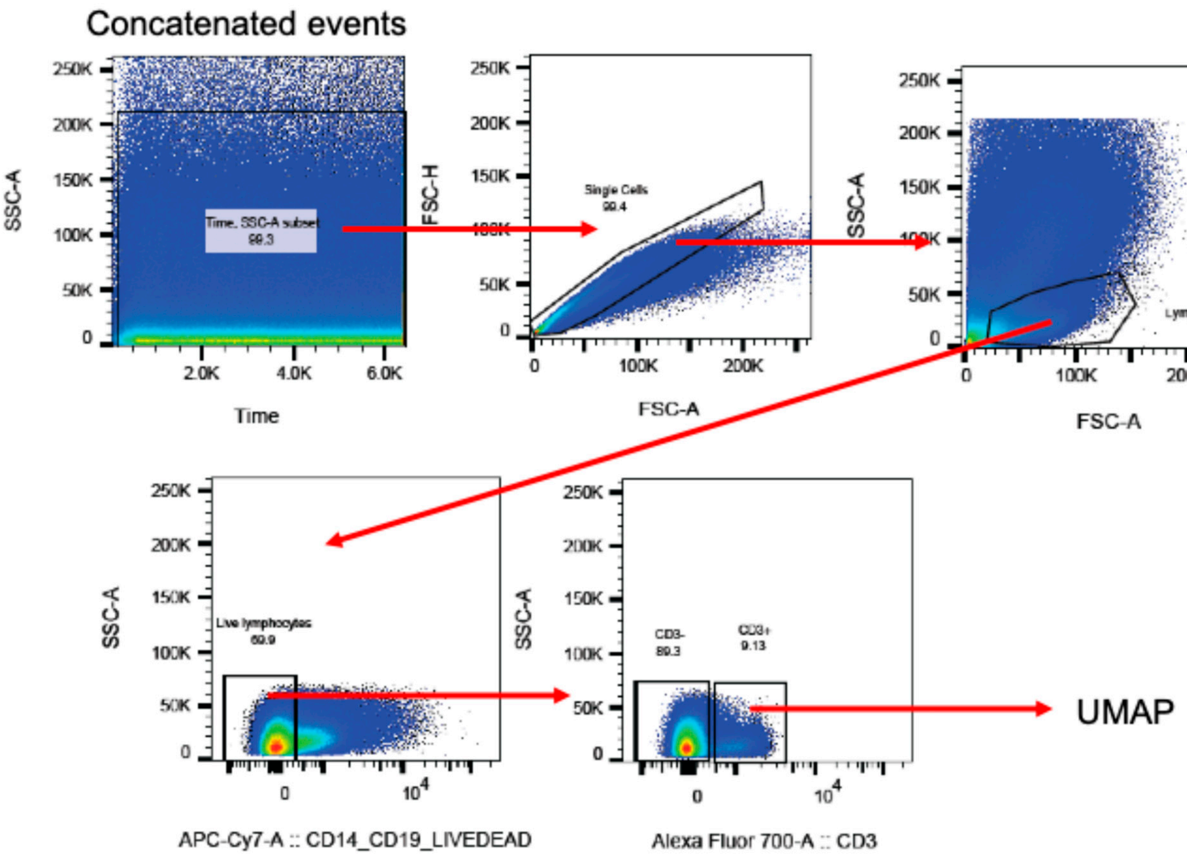


**Figure S2. Gating strategy and preprocessing workflow for high-dimensional analysis.** Flow cytometry data were initially subjected to quality control using the FlowAI algorithm to remove anomalous events. High-quality events from all samples were subsequently processed through sequential gating to select time-stable events, singlets, and lymphocytes based on light scatter properties. Dead cells, along with CD14⁺ and CD19⁺ cells, were excluded using a dump gate, and the remaining lymphocytes were separated into CD3⁺ and CD3⁻ compartments for downstream computational analysis. The resulting populations were exported for UMAP visualization and FlowSOM clustering.

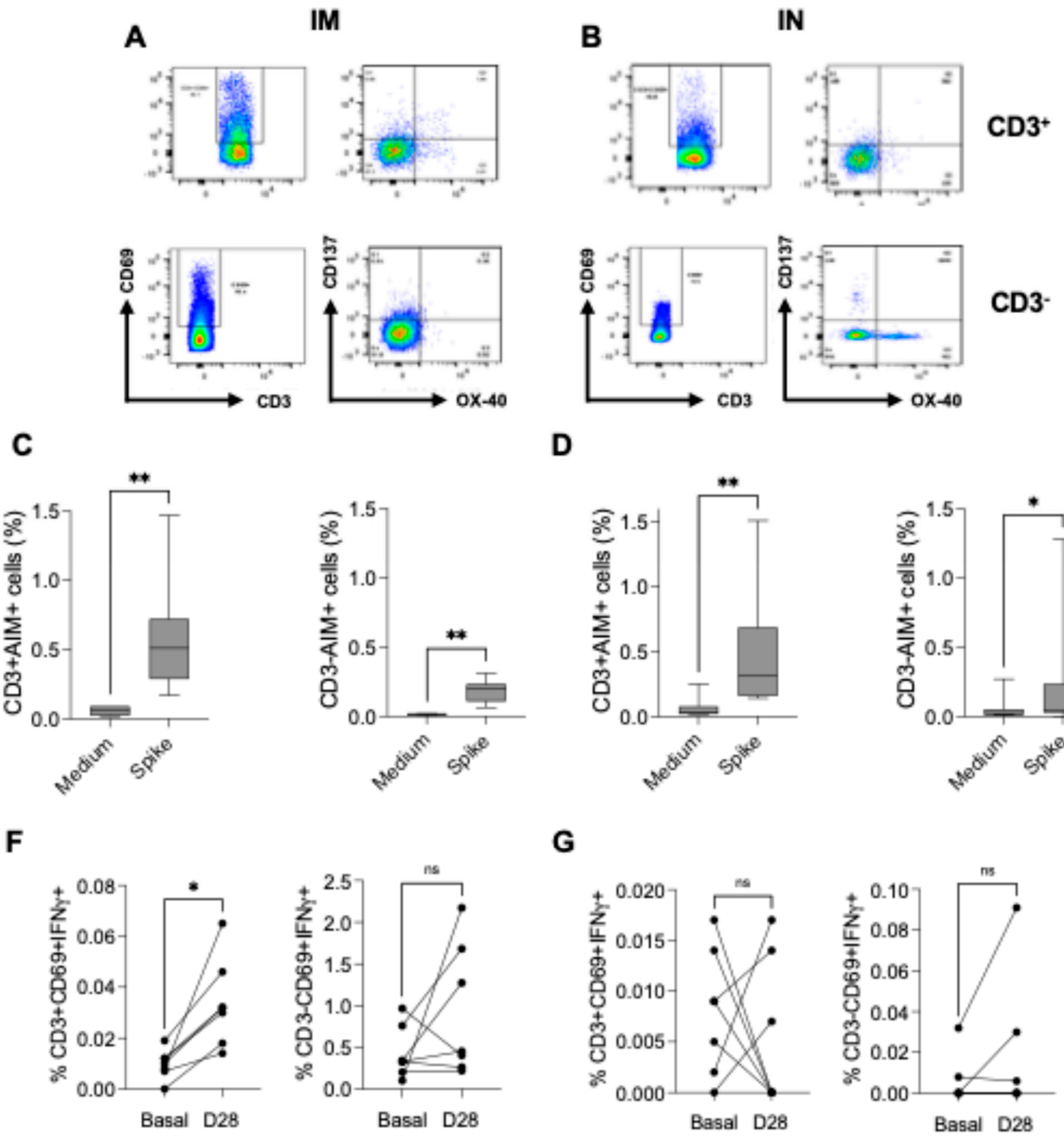


**Figure S3. Representative gating strategy and conventional AIM analysis of Spike-responsive $CD3^+$ and $CD3^-$ lymphocytes following AVX/COVID-12 vaccination.** Whole blood cells were stimulated in vitro with recombinant SARS-CoV-2 Wuhan Spike protein or culture medium alone for 18 h, and activation-induced marker (AIM) expression was evaluated by flow cytometry. (A,B) Representative gating strategy used to identify $AIM^+$ $CD4^+$ T cells ($CD69^+OX40^+$), $AIM^+$ $CD8^+$ T cells ($CD69^+CD137^+$), and activation-associated $CD3^-$ cells following intramuscular (IM) (A) or intranasal (IN) (B) vaccination. Frequencies of Spike-responsive $AIM^+$ $CD4^+$, $AIM^+$ $CD8^+$, and activation-associated $CD3^-$ cells (C, D) and IFN$\gamma$-producing cells (F, G) were measured 28 days after IM (C, F, n = 8) or IN (D, G, n = 9) vaccination. Data are presented as box plots showing the median and interquartile range, or as before-after comparisons at 28 days post-vaccination. Statistical comparisons were performed using the Mann–Whitney U test. $*p < 0.05$; $**p < 0.01$.
